# Pembrolizumab: First in Class for Treatment of Metastatic Melanoma

**DOI:** 10.6004/jadpro.2015.6.3.5

**Published:** 2015-05-01

**Authors:** Carrie Barnhart

**Affiliations:** Billings Clinic Cancer Center, Billings, Montana

Malignant melanoma accounts for only 2% of skin cancers, but it is the most deadly form of skin cancer. Melanoma rates have been increasing over the past 30 years. Melanoma is 10 to 20 times more common in Caucasians than in those of Hispanic or African descent. Metastatic (or stage IV) melanoma has a 5-year survival rate of 15% to 20% (American Cancer Society, 2014).

Pembrolizumab (Keytruda), a human programmed death receptor-1 (PD-1)–blocking antibody for intravenous infusion, was approved in September 2014 for the treatment of patients with unresectable or metastatic melanoma and disease progression following previous ipilimumab (Yervoy) and, if *BRAF V600* mutation–positive, a BRAF inhibitor (Merck, 2014a). *BRAF* mutations occur in 46% to 48% of patients with melanoma and are more likely to be found in younger patients and on intermittently sun-exposed tumors (Hall & Kudchadkar, 2014). However, resistance in *BRAF*-mutated melanoma appears to develop at 6 to 7 months (Long, Stroyakovskiy, & Gogas, 2014), so pembrolizumab may be an option upon recurrence (Robert et al., 2014).

Prior to 2011, US Food and Drug Administration (FDA)-approved treatment options for unresectable metastatic melanoma were limited to high-dose interleukin (IL-2), temozolomide, or dacarbazine, none of which showed a survival benefit (Middleton et al., 2000). The newer agents trametinib (Mekinist), ipili-mumab, and the BRAF inhibitors dabrafenib (Tafinlar) and vemurafenib (Zelboraf) have shown significant improvement in overall survival (see Table 1; Palathinkal, Sharma, Koon, & Bordeaux, 2014).

**Table 1:**
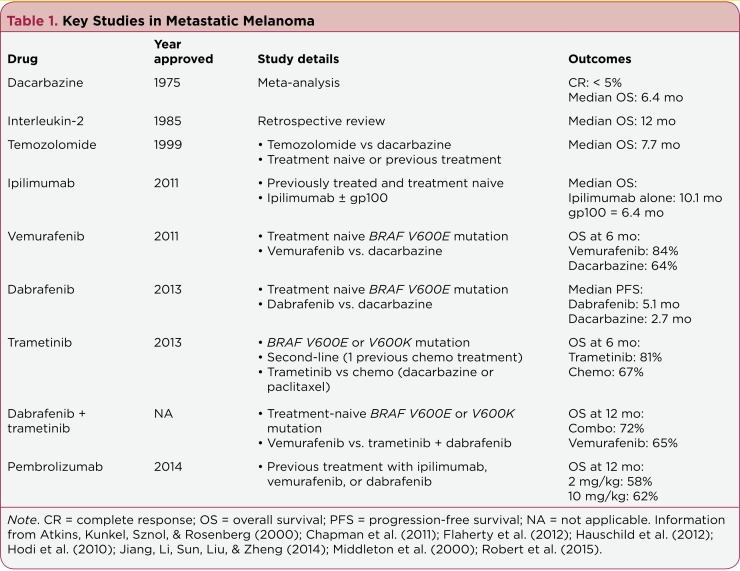
Key Studies in Metastatic Melanoma

One-year overall survival data with pembrolizumab have been reported at 58% for the 2-mg/kg dose and 62% for the 10-mg/kg dose (Robert et al., 2014). One-year survival rates for stage IV melanoma in 2009 were 33% to 62%. An elevated serum lactate dehydrogenase (LDH) level at the time of diagnosis was an independent and highly significant predictor of poor prognosis (Balch et al., 2009).

Previously known as MK-3475 or lambrolizumab, pembrolizumab is a first-in-class FDA-approved agent of a number of PD-1 or programmed death ligand (PD-L1) antibodies. Other drugs in this class include nivolumab (Opdivo) and the investigational agent MDPL-3280A (National Institutes of Health [NIH], 2014). The monoclonal antibodies in this class were considered "drugs of the year" in 2013 by European cancer researchers (Robert, Soria, & Eggermont, 2013).

## MECHANISM OF ACTION

In a nonmalignant activated T cell, PD-1 is an inhibitory receptor expressed on the surface to downregulate excessive immune responses. However, in malignant cells, it is hypothesized that PD-1 and one of its ligands, PD-L1, are responsible for a tumor cell’s ability to evade normal immune cell death. The ligands PD-L1 and PD-L2 are expressed on tumor cells. When PD-1 binds to PD-L1 or PD-L2, the activation causes immunosuppression and prevents the immune system from destroying the tumor cell (see Figure). Several agents such as ipilimumab, nivolumab, BMS-936559, and MDPL-3280A are now available or are being developed to provide immune checkpoint blockade (Ott, 2014).

**Figure 1:**
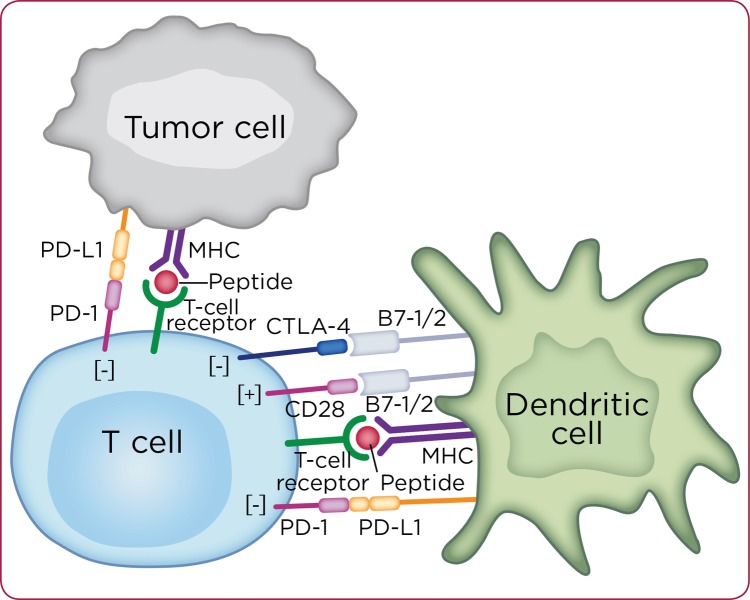
T-cell interaction with dendritic cells and tumor cells. CTLA4 = cytotoxic T-lymphocyte– associated protein 4; MHC = major histocompatibility complex; PD-1 = programmed death receptor-1; PD-L1 = programmed death ligand. Reprinted with permission from Ott (2014).

## CLINICAL STUDIES

Pembrolizumab was granted breakthrough therapy designation by the FDA because of early data showing benefit in patients with unresectable or metastatic melanoma. Pembrolizumab has been evaluated in a number of trials in metastatic melanoma (Hamid et al., 2013, 2014; Robert et al., 2014).

An expansion of the KEYNOTE-001 trial led to FDA approval of pembrolizumab. This was a multicenter, open-label, dose-comparative randomized phase IB trial of patients with unresectable or metastatic melanoma who had progressed after previous treatment. The study enrolled 173 patients, with the patient population having a median age of 61 years, an Eastern Cooperative Oncology Group (ECOG) performance scale of 0 or 1, and 40% females. Patients were randomized to receive pembrolizumab at either 2 or 10 mg/kg intravenously (IV) every 3 weeks until disease progression or unacceptable toxicity. All patients had disease progression after at least two doses of ipilimumab, and patients with *BRAF*-mutant melanoma were required to have previous treatment with at least one of the BRAF or MEK inhibitors (vemurafenib, dabrafenib, or trametinib). Seventy-three percent of patients had received two or more previous therapies for advanced or metastatic disease. The primary endpoint of the trial was overall response rate (ORR).

The ORR was similar in both arms at 24%. In the 2-mg/kg arm, 1 patient had a complete response, and 20 patients (24%) had a partial response. There were no complete responses in the 10-mg/kg group, and 32% of patients experienced a partial response. The estimated 1-year overall survival rate was 58% in the 2-mg/kg group and 63% in the 10-mg/kg group (Robert et al., 2014).

Pembrolizumab is also being studied in a number of tumor types, including non–small cell lung cancer, urothelial tract cancers, head and neck squamous cell cancers, gastric cancer, triple-negative breast cancer, colorectal cancer, and hematologic disorders (NIH, 2014). Pembrolizumab has shown promising results in PD-L1–positive bladder cancer in a phase IB study (Merck, 2014b).

## ADVERSE EVENTS

In the KEYNOTE-001 study, adverse events were similar for patients receiving either 2 mg/kg or 10 mg/kg of pembrolizumab. Although 82% of patients in the trial reported an adverse event, only 12% of patients had any grade 3 or 4 adverse event. Fatigue, pruritus, and rash were the most common adverse events. As most patients had received prior ipilimumab, investigators closely monitored patients receiving pembrolizumab for similar reactions. Grade 3 or 4 immune-mediated adverse reactions, such as autoimmune hepatitis, maculopapular rash, or pancreatitis, occurred in less than 0.02% of patients. Immune-mediated adverse events were treated with corticosteroids, with 0.02% of patients requiring permanent discontinuation of pembrolizumab (Robert et al., 2014). Anemia occurred in over 50% of patients, with 8% of all patients developing grade 3 or 4 anemia (Merck, 2014a).

Although rare, many of the immune-mediated adverse reactions occurred weeks to months after the administration of pembrolizumab. The pembrolizumab package insert includes six key warnings about immune-mediated precautions (Table 2). Immune-mediated pneumonitis occurred in about 3% of patients at a median 5 months into therapy and lasted 4.9 months. Patients with signs and symptoms of pneumonitis should have a chest x-ray or CT (computed tomography) scan to confirm the diagnosis and be given corticosteroids if grade 2 or higher. If grade 2 pneumonitis develops, pembrolizumab should be withheld until symptoms resolve. Pembrolizumab should be discontinued for severe or life-threatening (grade 3 or 4) pneumonitis (Merck, 2014a).

**Table 2:**
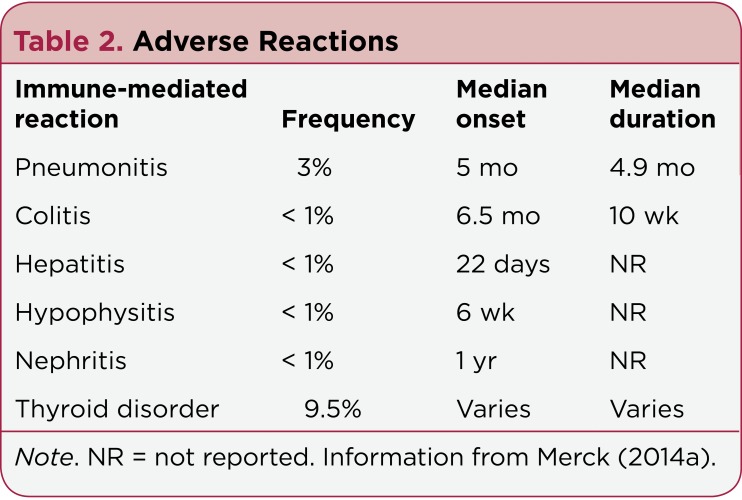
Adverse Reactions

Immune-mediated colitis, hepatitis, nephritis, and hypophysitis occurred in less than 1% of patients. Colitis started at a median of 6.5 months into therapy and lasted about 10 weeks. Hepatitis onset was at 22 days, hypophysitis occurred 6 weeks into therapy, and nephritis started nearly 1 year after initiation of therapy.

Patients being treated with pembrolizumab should be monitored for signs and symptoms of immune-mediated adverse reactions, and corticosteroids should be administered for grade 2 or greater reactions. Pembrolizumab should be withheld for moderate to severe symptoms (grade 3) and permanently discontinued for life-threatening conditions (grade 4). Immune-mediated thyroid disorders can occur at any time during treatment. Both hypo- and hyperthyroidism were seen, with hypothyroidism being more common. Hypothyroidism can be managed with replacement therapy. Grade 3 or 4 hyperthyroidism would necessitate withholding or discontinuing pembrolizumab (Merck, 2014a).

## DOSING AND ADMINISTRATION

Pembrolizumab is administered at a dose of 2 mg/kg IV every 3 weeks. It is administered as an IV infusion over 30 minutes, with a low-protein binding 0.2 to 5 µ in-line filter. The dose should be mixed in 0.9% sodium chloride to a final concentration of between 1 and 10 mg/mL. Treatment with pembrolizumab should continue until disease progression or unacceptable toxicity (Merck, 2014a).

Age, gender, renal impairment, mild hepatic impairment, obesity, and tumor burden had no clinically important effect on the clearance of pembrolizumab; therefore, no dosage adjustments are recommended (Merck, 2014a).

Most patients experiencing grade 1 events can be managed with supportive care and continued on pembrolizumab with close monitoring. A dose should be withheld and corticosteroids administered for most grade 2 or 3 toxicities, including pneumonitis, colitis, hypophysitis, nephritis, hyperthyroidism, elevated liver function tests, or other grade 2 or 3 treatment-related adverse events. Pembrolizumab can usually be resumed at provider discretion if the adverse reaction returns to grade 0 or 1 following appropriate steroid taper.

Pembrolizumab should be permanently discontinued if the patient has a severe (grade 4) adverse reaction, including infusion-related reactions, pneumonitis, nephritis, or aspartate aminotransferase (AST) or alanine aminotransferase (ALT) levels greater than five times the upper limit of normal (ULN), total bilirubin greater than three times ULN, failure of the adverse reaction to return to grade 0 or 1, recurrence of a grade 3 or higher event, or failure to taper steroids within 12 weeks of initiation (Merck, 2014a).

Pembrolizumab may cause fetal harm when administered to a pregnant woman. Thus, it is recommended for women of child-bearing potential to use highly effective contraception during pembrolizumab treatment and for 4 months after the last dose (Merck, 2014a).

## IMPLICATIONS FOR ONCOLOGY ADVANCED PRACTITIONERS

Routine lab monitoring of patients receiving pembrolizumab should include complete blood cell count with differential, chemistry panel, liver function tests, and thyroid-stimulating hormone. Patient education and monitoring are crucial to recognize rare but potentially serious immune-mediated adverse reactions. Because many of these adverse events occur months after initiation of pembrolizumab therapy, it is important to continue monitoring patients even after systemic therapy has been discontinued.

The National Comprehensive Cancer Network (NCCN) guidelines currently recommend pembrolizumab, nivolumab, ipilimumab, or high-dose IL-2 for first-line therapy for metastatic or unresectable BRAF V600 wild-type melanoma (NCCN, 2015).

Pembrolizumab offers a new therapeutic option for patients who have already received systemic treatment for metastatic melanoma yet their disease progressed.
